# A PET-CT study on neuroinflammation in Huntington’s disease patients participating in a randomized trial with laquinimod

**DOI:** 10.1093/braincomms/fcad084

**Published:** 2023-04-03

**Authors:** Andreas-Antonios Roussakis, Marta Gennaro, Mark Forrest Gordon, Ralf Reilmann, Beth Borowsky, Gail Rynkowski, Nicholas P Lao-Kaim, Zoe Papoutsou, Juha-Matti Savola, Michael R Hayden, David R Owen, Nicola Kalk, Anne Lingford-Hughes, Roger N Gunn, Graham Searle, Sarah J Tabrizi, Paola Piccini

**Affiliations:** Brain Sciences, Imperial College London, Hammersmith Hospital, London W12 0NN, UK; Brain Sciences, Imperial College London, Hammersmith Hospital, London W12 0NN, UK; Teva Pharmaceuticals, West Chester, PA 19380, USA; George-Huntington-Institute, Münster 48149, Germany; Teva Pharmaceuticals, West Chester, PA 19380, USA; Teva Pharmaceuticals, West Chester, PA 19380, USA; Brain Sciences, Imperial College London, Hammersmith Hospital, London W12 0NN, UK; Brain Sciences, Imperial College London, Hammersmith Hospital, London W12 0NN, UK; Teva Pharmaceuticals International GmbH, 4051 Basel, Switzerland; Centre for Molecular Medicine and Therapeutics, BC Children’s Hospital and Research Institute, University of British Columbia, Vancouver V5Z 4H4, Canada; Brain Sciences, Imperial College London, Hammersmith Hospital, London W12 0NN, UK; Brain Sciences, Imperial College London, Hammersmith Hospital, London W12 0NN, UK; Brain Sciences, Imperial College London, Hammersmith Hospital, London W12 0NN, UK; Brain Sciences, Imperial College London, Hammersmith Hospital, London W12 0NN, UK; Invicro, Hammersmith Hospital,, London W12 0NN, UK; Invicro, Hammersmith Hospital,, London W12 0NN, UK; Huntington’s Disease Centre, Department of Neurodegenerative Disease, UCL Queen Square Institute of Neurology, London WC1N 3BG, UK; Brain Sciences, Imperial College London, Hammersmith Hospital, London W12 0NN, UK

**Keywords:** microglia, Huntington’s, TSPO, ^11^C-PBR28, laquinimod

## Abstract

Microglia activation, an indicator of central nervous system inflammation, is believed to contribute to the pathology of Huntington’s disease. Laquinimod is capable of regulating microglia. By targeting the translocator protein, ^11^C-PBR28 PET-CT imaging can be used to assess the state of regional gliosis *in vivo* and explore the effects of laquinimod treatment. This study relates to the LEGATO-HD, multi-centre, double-blinded, Phase 2 clinical trial with laquinimod (US National Registration: NCT02215616). Fifteen patients of the UK LEGATO-HD cohort (mean age: 45.2 ± 7.4 years; disease duration: 5.6 ± 3.0 years) were treated with laquinimod (0.5 mg, *N* = 4; 1.0 mg, *N* = 6) or placebo (*N* = 5) daily. All participants had one ^11^C-PBR28 PET-CT and one brain MRI scan before laquinimod (or placebo) and at the end of treatment (12 months apart). PET imaging data were quantified to produce ^11^C-PBR28 distribution volume ratios. These ratios were calculated for the caudate and putamen using the reference Logan plot with the corpus callosum as the reference region. Partial volume effect corrections (Müller–Gartner algorithm) were applied. Differences were sought in Unified Huntington’s Disease Rating Scale scores and regional distribution volume ratios between baseline and follow-up and between the two treatment groups (laquinimod versus placebo). No significant change in ^11^C-PBR28 distribution volume ratios was found post treatment in the caudate and putamen for both those treated with laquinimod (*N* = 10) and those treated with placebo (*N* = 5). Over time, the patients treated with laquinimod did not show a significant clinical improvement. Data from the ^11^C-PBR28 PET-CT study indicate that laquinimod may not have affected regional translocator protein expression and clinical performance over the studied period.

## Teaser

This is a longitudinal study in Huntington’s disease with laquinimod (microglia modulator) and ^11^C-PBR28 PET (specific marker of reactive gliosis). Roussakis and Gennaro report that 12-month long laquinimod treatment (0.5 or 1 mg daily versus placebo) does not affect regional microglia activation.

## Graphical Abstract

**Graphical Abstract:**
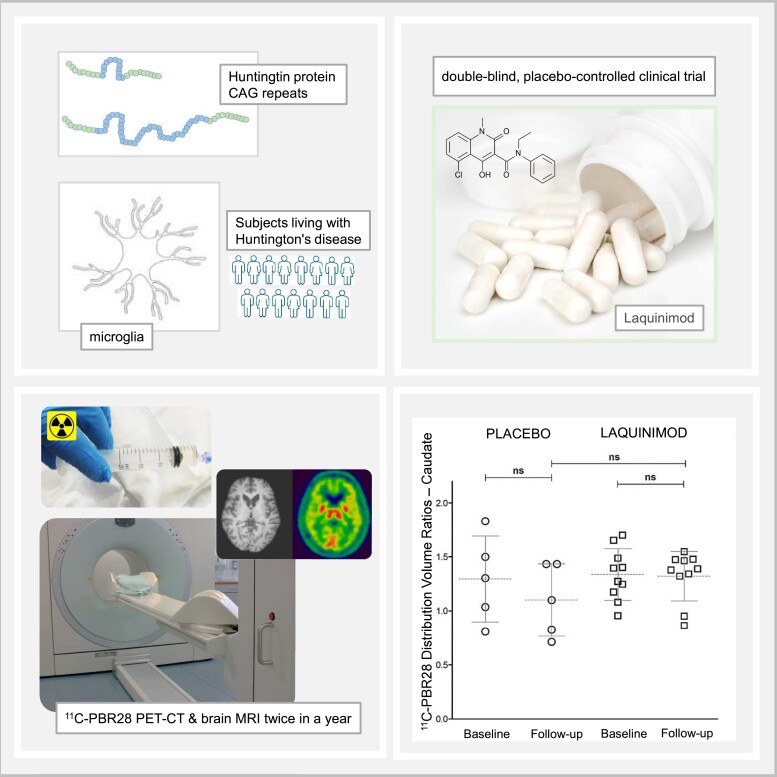

## Introduction

Over past decades, clinical studies in Huntington’s disease have largely focused on understanding the pathology of neuronal damage. Indeed, all patients with Huntington’s disease develop a plethora of alterations in the brain at both structural and functional levels, primarily in the striatum, as a result of a CAG repeat expansion on Chromosome 4.^1–3^ With current evidence, however, standard therapeutics do not restore brain damage or modify Huntington’s disease progression.^4^

In the human brain, microglia become ‘activated’ in response to neuronal damage and loss. This process is complicated and can become chronic. Many of the features of reactive gliosis are shared by the most common neurodegenerative disorders including Parkinson’s^5^ and Alzheimer’s diseases.^6,7^ Microglia activation is considered an indicator of central nervous system inflammation.^7,8^ In the context of Huntington’s disease, microglia activation is believed to contribute to the disease pathology. Neurons affected by clusters of abnormal Huntingtin are progressively damaged. It is believed that reactive microglia fail to address regional damage adequately over time. As Huntington’s disease progresses, regional gliosis becomes a toxic environment for the damaged neurons. These points enable microglia as a target for therapeutic intervention in Huntington’s disease and a promising area for research.

The current study concerns the imaging outcomes of a multi-centre, randomized, double-blind, placebo-controlled, parallel-group clinical trial in Huntington’s disease. On 0.5 and 1.0 mg daily, laquinimod has shown favourable CNS kinetics and good tolerability in healthy volunteers and multiple sclerosis patients.^9–11^ To evaluate microglia activation *in vivo*, we employ ^11^C-PBR28, a second-generation PET radioligand that is highly specific for the translocator protein (TSPO).^12–14^

The TSPO is expressed at various sites in the human CNS, including the mitochondria of microglia.^15^ When the TSPO is in high density within microglia, it is believed to reflect regional gliosis.^16–18^ The chosen brain regions for this PET study were caudate and putamen, as they are predominantly involved in motor control. The secondary objective of this PET study was to look at a possible relation of striatal ^11^C-PBR28 binding to the clinical characteristics of Huntington’s disease patients.

## Materials and methods

### Participants, eligibility and regulatory approvals

The main clinical trial was organized by Teva Pharmaceuticals in collaboration with the Huntington Study Group and the European Huntington’s Disease Network. The clinical trial details can be found online at the US National Register (ClinicalTrials.gov: NCT02215616) and the EU Drug Regulating Authorities Clinical Trials Database (EudraCT: 2014-000418-75). For the UK sites, the study was approved by the Health Research Authority (IRAS: 151325), the South Central Hampshire B research ethics committee (REC Reference: 14/SC/1340), the Medicines and Healthcare Regulatory Agency (MHRA: 34261/0017/001) and the Administration of Radioactive Medicinal Products Committee (ARSAC: 630/3764/32453), UK. Enrolment into the PET-CT imaging study was optional. Written consent was sought in accordance with the Declaration of Helsinki. Dynamic randomization (1:1:1) was performed with interactive response technology to balance the treatment groups at the UK centres. The research team at Imperial College London and the recruiting UK sites as well as the study participants were blinded for the duration of the treatment period.

Participants enrolled in the LEGATO-HD trial (Sponsor’s reference: TV5600-CNS-20007) were referred to Imperial College London for PET imaging. Nineteen Huntington’s disease patients met the inclusion and exclusion criteria (see Supplementary Table 1) of the PET protocol and successfully passed screening. Two patients withdrew and did not complete the PET study. One subject had a baseline MRI but no baseline PET-CT and was not eligible for the follow-up. One follow-up PET-CT scan failed technically during quality control (QC) testing. In summary, 15 participants completed the PET imaging study with PET-CT and brain MRI at baseline and follow-up (∼12 months apart). The mean interval between baseline and end-of-treatment PET-CT imaging was 0.95 years (±0.03 SD) for all patients (*N* = 15) who completed the treatment period.

In addition, we included PET-CT and MRI data from a group of 21 healthy volunteers. Details about this dataset can be found elsewhere.^19,20^ All healthy volunteers had one PET-CT and one brain MRI scan at the Imanova Imaging Centre under the same protocol of Huntington’s disease patients. Analyses of this data set were performed at outlined below (see relevant section).

Huntington’s disease patients (all had confirmed CAG repeats >40) were administered laquinimod (0.5 or 1.0 mg) or placebo on a daily basis and were reviewed by Huntington’s disease specialist clinical teams at regular intervals. The severity of Huntington’s disease was assessed using the Unified Huntington’s Disease Rating Scale (UHDRS).^21^ UHDRS-Motor and UHDRS-Functional subscores were calculated for each patient from ‘Motor Assessment’ and ‘Functional Capacity’ components of the form. Subjects on high doses of diazepam (20-30 mg/daily) were excluded from the PET-CT imaging study as this may have had central TSPO binding effects,^22^ thus potentially compromising the quality of the data. At enrolment for the PET study, we excluded those Huntington’s disease patients who were treated with benzodiazepines, tetrabenazine, neuroleptics, or *N*-methyl-D-aspartate receptor blocking agents. We did not screen for non-steroidal anti-inflammatory drugs.

The *rs6971* locus in the TSPO gene has a polymorphism that affects the binding of all known second generation TSPO-specific radioligands.^23–25^ In the general population, people without the polymorphism have high affinity binding for PBR28, homozygotes have low affinity binding and heterozygotes express both high and low affinity for it (mixed affinity binding). In this study, we recruited high (HAB) and mixed (MAB) affinity binders. For women of childbearing potential, negative urine pregnancy testing was required at the PET imaging facility prior to any procedure involving radiation. These tests were in addition to the standard safety measures taken during the main LEGATO-HD study.

### Data acquisition and data management

All ^11^C-PBR28 PET-CT imaging took place at the Imanova (currently Invicro Ltd) imaging centre at the Hammersmith Hospital site in London, UK. Specific activity, chemical composition and radiochemical purity of ^11^C-PBR28 were determined by radioactive high-performance liquid chromatography coupled with a gamma detector. Further details on radioligand synthesis can be found here.^26,27^

^11^C-PBR28 was injected as an intravenous bolus injection in the PET suite followed by a 90-min dynamic acquisition. The mean ^11^C-PBR28 injected activity was 323.3 ± 38.5 MBq. PET-CT scanning took place on a Siemens Biograph Hi-Rez 6 PET-CT scanner (Siemens Healthcare, Erlangen, Germany Ltd). PET data were reconstructed using a filtered back projection algorithm (direct inversion Fourier transform) with a 128 matrix, zoom of 2.6, a transaxial Gaussian filter of 5 mm producing images with an isotropic voxel size of 2 × 2 × 2 mm^3^. Attenuation and scatter correction were applied based on the low-dose CT data. The dynamic images were binned into 26 frames (8 × 15, 3 × 60, 5 × 120, 5 × 300 and 5 × 600 s).

All patients had a volumetric T_1_-weighted MR sequence at baseline and at follow-up (end of treatment or early termination) within 4 weeks of each PET-CT scan. All healthy volunteers had a volumetric T_1_-weighted MR sequence. T_1_-weighted magnetization-prepared rapid acquisition gradient echo (MPRAGE) images were acquired under a standardized protocol across the recruiting sites as described before.^28^ MPRAGE data were used for the volumetric analyses and for co-registration with the PET images.

The imaging analysis team was given access to view and electronically download the MRI data in digitized pseudonymized form via the trial-specific online platform. MRI scans of the Huntington’s disease patients were in DICOM (digital imaging and communications in medicine) format, without any preprocessing, and labelled with a pseudonymized code identical to that given to each individual patient. Raw MRI data were matched to the PET-CT imaging data, also saved in DICOM format by the imaging analysis team. QC testing was performed at the MR and PET-CT facilities where data had been acquired and at a second level at the PET-CT imaging workstation at Imperial College London.

### Imaging data analyses

Processing and kinetic modelling of the ^11^C-PBR28 PET-CT and MPRAGE MRI data were performed using an in-house integrated pipeline as described before^29^ with MIAKAT™ (molecular imaging and kinetic analysis toolbox; Invicro Imaging Centre, London, UK) software for academic use.^30^ MIAKAT is implemented in MATLAB® (version 2017b; Mathworks, Natick, MA, USA), SPM12 (Statistical Parametric Mapping version 12; Wellcome Trust Centre for Neuroimaging, London, UK) and FSL (version 5.0.10; FMRIB Image Analysis Group, Oxford, UK).

MPRAGE images were rigidly registered to the Montreal Neurological Institute space after brain extraction and segmentation. Binary masks of the caudate and putamen, referred to as region of interest (ROIs), and the corpus callosum (CC) were created on the FreeSurfer images analysis suite (https://surfer.nmr.mgh.harvard.edu) using individual MPRAGE and then customized manually on Analyze (version 11.0; Biomedical Imaging Resource, Mayo Clinic) to account for segmentation inaccuracies due to atrophy. Frame-by-frame motion correction was applied to the dynamic PET data. A summed PET image was created and co-registered to ROIs, CC binary masks and the CIC atlas^31^ using normalized information as the cost function. ^11^C-PBR28 time activity curves were generated. The distribution volume ratio (DVR) values where calculated employing the Logan reference kinetic plot^32^ using the CC as a pseudo-reference region. All steps of the above analysis pipeline successfully passed QC testing. MRI volumetric assessment was performed using FreeSurfer with a volume-based subcortical approach as previously reported.^33^

Partial volume effect correction (PVC) was applied to the PET data, using the PET PVE12 SPM toolbox,^34^ to account for Huntington’s disease-related cerebral atrophy. White and grey matter binary masks were created with FreeSurfer and subsequently used as references in the Müller-Gärtner three-compartmental algorithm^35^ with a frame-by-frame approach on the dynamic PET images.

### Statistical analyses

Statistical analyses were performed using the IBM SPSS® Statistics software, Version 25 for Microsoft windows. The significance (alpha) level was set at *α* = 0.05. Homogeneity of variances was performed with Bartlett’s test. The Kolmogorov-Smirnov normality test was performed to examine if variables were normally distributed. Graph illustrations were performed using the GraphPad Prism software, Version 6 for Microsoft windows and Microsoft Office Excel.

Validation of the Huntington’s disease data set was made through comparisons of means between the Huntington’s disease patients (baseline data) and those from the group of healthy volunteers. Comparisons of caudal and putaminal ^11^C-PBR DVRs and volumes (MRI data) as well as of age between the patients and healthy volunteers were performed with *t*-test for independent samples. Chi-squared (*χ*^2^) test was performed to assess gender differences, followed by Yates’s correction. For the Huntington’s disease group, we sought for correlations of regional ^11^C-PBR DVRs with CAG repeats, age, disease duration from diagnosis (DD_diagn_), disease duration from onset (DD_onset_) and UHDRS scores using non-parametric Spearman’s rho coefficient (*r*). Benjamini-Hochberg procedure was applied afterwards.

Huntington’s disease patients were then divided into two groups: placebo and laquinimod (either 0.5 or 1.0 mg) treatment. Comparisons of caudal and putaminal ^11^C-PBR28 DVRs and volumes (MRI data) between baseline and follow-up were performed with paired *t*-tests. Comparisons of ^11^C-PBR28 DVRs between placebo and laquinimod treatment groups at baseline were performed with non-parametric Mann-Whitney *U* tests. Comparisons between baseline and follow-up ^11^C-PBR28 DVRs and UHDRS scores were performed with non-parametric Wilcoxon signed-rank test for related samples. Change over time is shown with ^11^C-PBR28 ΔDVRs. *│*Δ*│* = *│* (Follow-up DVR) – (Baseline DVR)*│*. Percentage DVR changes were calculated as % DVR change = 100 × *│*Δ*│*/(Baseline DVR).

## Results

**Figure 1:**
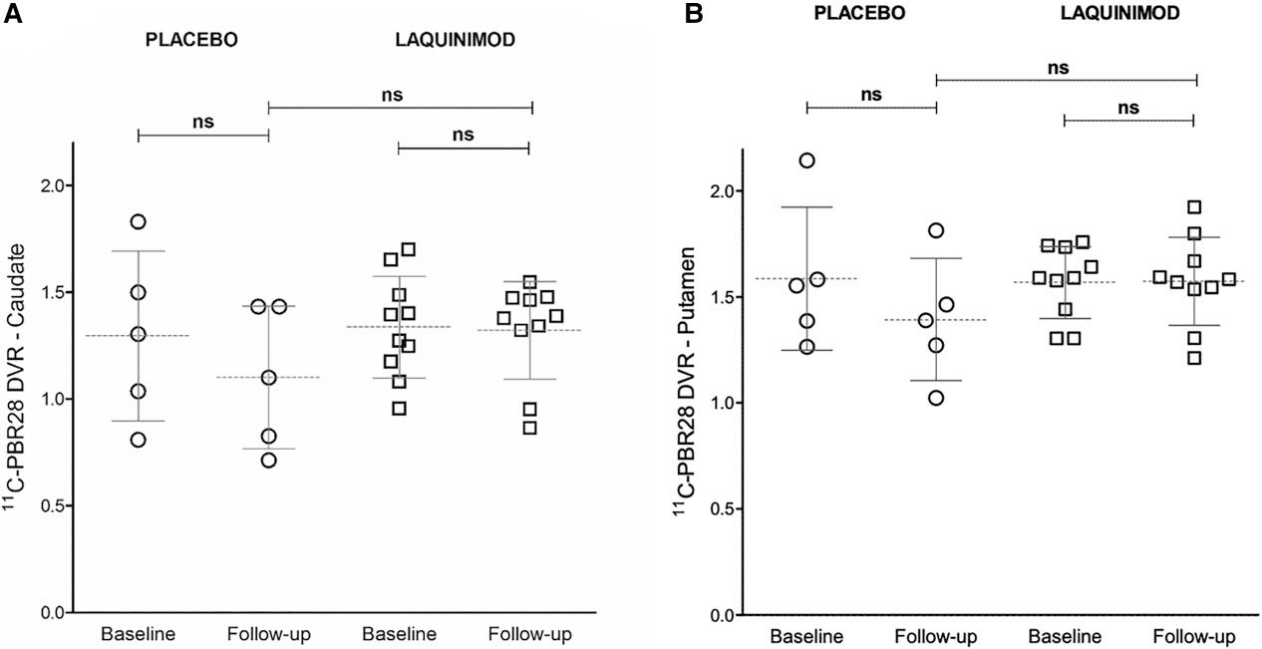
**PET data—placebo and laquinimod (combined).** Scatter plot of ^11^C-PBR DVR values for caudate (**A**) and putamen (**B**). Circles = Huntington’s disease patients treated with placebo (*N* = 5); squares = Huntington’s disease patients treated with laquinimod (all doses, *N* = 10). Comparison between baseline and follow-up DVR values was made with paired *t*-tests. Comparisons of DVRs between placebo and laquinimod treatment groups at follow-up was performed with non-parametric Mann-Whitney U-tests.

**Figure 2:**
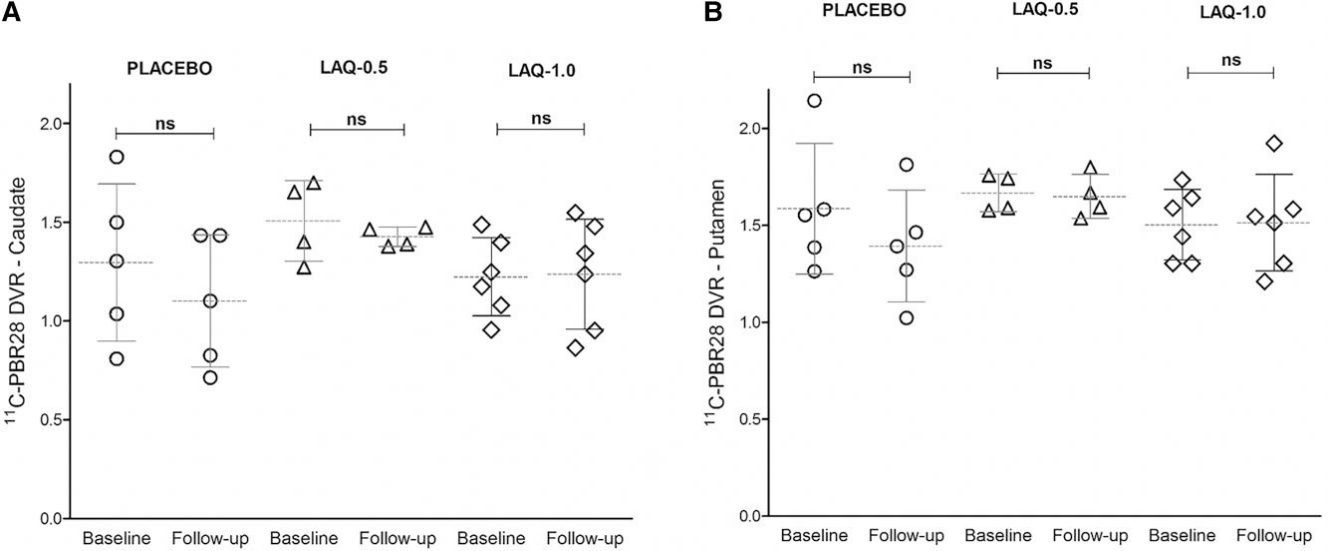
**PET data—placebo and laquinimod (two dose groups).** Scatter plot of ^11^C-PBR DVR values for caudate (**A**) and putamen (**B**). Circles = Huntington’s disease patients treated with placebo (*N* = 5); triangles = Huntington’s disease patients treated with Laquinimod 0.5 mg (*N* = 4); rhombi = Huntington’s disease patients treated with Laquinimod 1.0 mg (*N* = 6). Comparison between baseline and follow-up DVR values was made with paired *t*-tests.

Randomization (1:1:1) of Huntington’s disease patients resulted in three groups: laquinimod 0.5 mg (*N* = 4), laquinimod 1.0 mg (*N* = 6) and placebo (*N* = 5). Imaging (PET and MRI) and clinical data are tabulated below (see Tables 1–3 and Figs 1 and 2) and as Supplementary material (Supplementary Table 2 and Figs 1–6). Uncorrected DVR values are shown in Table 2.

**Table 1:** Imaging data: Huntington’s disease versus healthy volunteers

PET data (baseline)—Huntington’s disease patients versus healthy volunteers		
	Patients (*N* = 18)	Healthy volunteers (*N* = 21)
TSPO affinity	7MAB:11HAB	10MAB:11HAB
Age (years)	44.39 ± 2.75	39.38 ± 12.96 ns^a^
^11^C-PBR28 caudate DVR	1.35 ± 0.26	0.83 ± 0.15^***,a^
^11^C-PBR28 putamen DVR	1.59 ± 0.24	1.05 ± 0.17^***,a^

Volumetric MRI data set—Huntington’s disease patients versus healthy volunteers			
	Patients (*N* = 19)	Patients (*N* = 16)	Healthy volunteers (*N* = 21)
	Baseline	Follow-up	Baseline
Caudate volume	3701 ± 932	3867 ± 1139 ns^b^	7671 ± 842^***,a^
Putamen volume	5368 ± 1204	5537 ± 1381 ns^b^	10 268 ± 859^***,a^

Values are given as mean ± 1 SD. Volumes are summed for left and right regions and rounded. Volume figures refer to mm^3^. DVR, distribution volume ratio (unitless); TSPO, translocator protein; ns, non-significant. ***Statistical significance *P* < 0.001. ^a^Comparison between baseline data of patients and healthy volunteers. ^b^Comparison between follow-up and baseline (patients only, *N* = 16).

**Table 2:** Huntington’s disease patients: laquinimod versus placebo

Treatment arm	Laquinimod (*N* = 10)		Placebo (*N* = 5)	
Gender	6M:4F ns		3M:2F	
CAG repeats	>40		>40	
TSPO affinity	4MAB:6HAB		2MAB:3HAB	
Interval between PET scans	0.93 ± 0.03 years		0.96 ± 0.02 years	

	Baseline	Follow-up	Baseline	Follow-up
Age (years)	47.58 ± 3.80	48.52 ± 3.79	43.95 ± 8.37 ns^a^	44.91 ± 8.37
DD_diagn_ (years)	3.89 ± 4.11	4.82 ± 4.11	4.50 ± 3.13 ns^a^	5.47 ± 3.12
DD_onset_ (years)	5.99 ± 3.85	6.99 ± 3.85	5.40 ± 2.37 ns^a^	6.41 ± 2.36
UHDRS-TMS	31.00 ± 9.53	31.00 ± 13.55 ns^b^	29.60 ± 14.73 ns^a^	25.70 ± 9.75 ns^c^
UHDRS-TFC	36.60 ± 4.59	34.40 ± 5.71 ns^b^	36.10 ± 3.51 ns^a^	36.10 ± 3.59 ns^c^
^11^C-PBR28 caudate DVR	1.34 ± 0.23	1.32 ± 0.23 ns^b^	1.30 ± 0.36 ns^a^	1.10 ± 0.33 ns^c^
^11^C-PBR28 putamen DVR	1.57 ± 0.16	1.57 ± 0.21 ns^b^	1.63 ± 0.38 ns^a^	1.37 ± 0.29 ns^c^
^11^C-PBR28 caudate DVR (uncorrected)	1.29 ± 0.13	1.31 ± 0.08 ns^b^	1.24 ± 0.18	1.20 ± 0.29 ns^c^
^11^C-PBR28 putamen DVR (uncorrected)	1.92 ± 0.19	1.93 ± 0.18 ns^b^	1.89 ± 0.28	1.80 ± 0.24 ns^c^

DD_diagn_, disease duration from diagnosis; DD_onset_, disease duration from onset; DVR, distribution volume ratio; TFC, total function capacity; TMS, total motor scores; UHDRS, Unified Huntington’s Disease Rating Scale; ns, non-significant. ^a^ Between baseline placebo and baseline laquinimod. ^b^ Between follow-up laquinimod and baseline laquinimod. ^c^Between follow-up placebo and baseline placebo.

**Table 3:** Huntington’s disease patients: laquinimod dose effect

Treatment arm	Laquinimod 0.5 mg (*N* = 4)		Laquinimod 1.0 mg (*N* = 6)	
Gender	2M:2F		4M:2F ns	
CAG repeats	>40		>40	
TSPO affinity	3MAB:1HAB		1MAB:5HAB	

	Baseline	Follow-up	Baseline	Follow-up
Age	49.52 ± 2.59	50.48 ± 2.60	40.24 ± 8.82 ns^a^	41.21 ± 8.83
DD_diagn_	5.21 ± 2.56	6.16 ± 2.55	4.03 ± 3.38 ns^a^	5.00 ± 3.37
DD_onset_	7.31 ± 1.68	8.32 ± 1.68	4.12 ± 1.84 ns^a^	5.14 ± 1.84
UHDRS-TMS	38.75 ± 14.46	28.75 ± 7.22 ns^b^	23.50 ± 11.37	23.67 ± 10.64 ns^c^
UHDRS-TFC	34.00 ± 1.58	36.50 ± 0.50 ns^b^	37.70 ± 3.73	35.83 ± 4.60 ns^c^
^11^C-PBR28 caudate DVR	1.51 ± 0.18	1.43 ± 0.04 ns^b^	1.22 ± 0.18	1.24 ± 0.28 ns^c^
^11^C-PBR28 putamen DVR	1.67 ± 0.08	1.65 ± 0.10 ns^b^	1.50 ± 0.17	1.51 ± 0.25 ns^c^

DD_diagn_, disease duration from diagnosis; DD_onset_, disease duration from onset; DVR, distribution volume ratio; TFC, total function capacity; TMS, total motor scores; UHDRS, Unified Huntington’s Disease Rating Scale; ns, non-statistically significant. ^a^ Between baseline 1.0 mg and baseline 0.5 mg. ^b^ Between follow-up 0.5 mg and baseline 0.5 mg. ^c^ Between follow-up 1.0 mg and baseline 1.0 mg.

### At baseline

Baseline ^11^C-PBR28 DVRs were significantly higher in the caudate [*P* < 0.001, *F* = 5.30, *t* = −7.54, 95% confidence interval (CI) = −0.66, −0.38] and the putamen (*P* < 0.001, *F* = 0.22, *t* = −8.15, 95% CI = −0.67, −0.40) in the Huntington’s disease group when compared with healthy volunteers. All patients showed marked striatal volume losses when compared with healthy volunteers at baseline (caudate: *P* < 0.001, *F* = 2.28, *t* = −5.74, 95% CI = −4622, −2667; putamen: *P* < 0.001, *t* = −7.0, *F* = 3.75, 95% CI = −5764, −3745). At enrolment, the healthy volunteers were age-matched to the Huntington’s disease group. However, not matched for gender (*χ*^2^ test = 4.14, post Yates’s correction; *P* < 0.05). See more in Tables 1 and 2, and Supplementary Figs 1 and 2.

^11^C-PBR DVRs correlated with the CAG repeats in 18 Huntington’s disease patients (caudate: *r* = 0.51, *P* < 0.05; putamen: *r* = 0.47, *P* < 0.05). DVR values also correlated with DD_diagn_ (caudate: *r* = 0.74, *P* < 0.001; putamen: *r* = 0.81, *P* < 0.001) and DD_onset_ (caudate: *r* = 0.65, *P* < 0.001; putamen: *r* = 0.67, *P* < 0.001; see Supplementary Figs 3 and 4). No correlation was found between regional ^11^C-PBR DVRs, age and the clinical severity scores.

The placebo group was not significantly different (based on statistical comparisons of age, DD_diagn_ and DD_onset_ at baseline) from the group treated with laquinimod (see Table 2). The laquinimod 0.5 mg subgroup was not significantly different at baseline from the group that was treated with 1.0 mg (see Table 3).

### Over time

At follow-up, the Huntington’s disease patients who were on placebo did not have a statistically significant change from baseline in their ^11^C-PBR28 DVRs in either the caudate or the putamen. At the end of the laquinimod treatment, the 10 Huntington’s disease patients did not show a statistically significant change in their regional ^11^C-PBR28 DVRs for either the caudate or the putamen. In this study, Huntington’s disease patients did not demonstrate a statistically significant change in volumes over time for either the caudate or the putamen. Patients treated with laquinimod did not show a statistically significant change at the end of treatment in the UHDRS severity motor and functional scores. No statistically significant change was seen in the placebo group in the UHDRS scores over time (see Tables 1 and 2, and Fig. 1). |Δ|DVRs and percentage DVR changes are shown in Supplementary Table 2 (see also Supplementary Figs 5 and 6).

Individually, each laquinimod treatment subgroup did not change significantly over time. Details for each laquinimod subgroup are shown in Table 3 and Fig. 2.

## Discussion

The primary aim of this PET-CT imaging study was to assess changes in the state of striatal microglia in Huntington’s disease due to treatment with laquinimod. Over 1 year, regional TSPO expression (reflected by ^11^C-PBR28 PET-CT DVRs) was different in the brains of Huntington’s disease participants. This change, however, was not statistically significant for patients treated with either placebo or laquinimod. At the end of the treatment period, Huntington’s disease patients (either on laquinimod or placebo) had no significant change from baseline in their clinical scores, while all maintained marked striatal volume losses (see Results and Table 1).

Considering that Huntington’s disease is a rare disease, we appreciate that this PET study may have been underpowered. We are aware that the duration of the follow-up period may not be ideal to detect significant changes in TSPO expression over time. This PET study is explorative and the findings need careful consideration before safe conclusions can be drawn about the potential of laquinimod to affect microglia activation *in vivo*.

At first, we would like to acknowledge that there is no consensus in the literature on how to best study neuroinflammation in Huntington’s disease *in vivo.*^36^ Despite the methodological differences with first-generation TSPO-specific binding studies, and ^11^C-PBR28 PEC-CT protocols for healthy volunteers and other disorders, our baseline PET results support a significant value of ^11^C-PBR28 PECT-CT for *in vivo* imaging of TSPO and for differentiating Huntington’s disease patients from healthy volunteers. In our study, baseline ^11^C-PBR28 DVRs in the caudate and putamen were significantly higher, and volumes were significantly lower, in the Huntington’s disease group when compared with healthy volunteers. The baseline DVR values correlated strongly with the number of CAG repeats (associated with high probability for severe disease), and with disease duration measures (DD_diagn_ and DD_onset_). These results are in line with previous work on manifest and pre-manifest Huntington’s disease,^37,38^ and add validity to the chosen methods.

For the purposes of this study, our Huntington’s participants make a representative group of Huntington’s disease patients. Our patients demonstrate variable disease durations and variable number of CAG repeats. Taking into account feasibility aspects (rarity of Huntington’s disease, difficulty of patients with manifest movement disorders to stay still for 90 min for a scan, risks associated with arterial cannulation), and results from previous ^11^C-PBR28 PEC-CT methodology studies,^14,39^ we decided to omit arterial cannulation and have a pragmatic approach at enrolment. We acknowledge that this approach may not be adequate for a study on a common disorder.

In the absence of arterial input function from the present design, quantification of TSPO-specific PET-CT data from patients with Huntington’s disease and marked atrophy required extra steps.^40,41^ By reviewing former research work on PBR28, TSPO expression and Huntington’s disease pathology, we decided to use the CC as the reference region after exploring several brain regions, including the cerebellum.^29^

Issues with TSPO imaging refer to variability in the TSPO affinity status and the integrity of the brain tissue under study. In Huntington’s disease, post-mortem data demonstrate the presence of reactive microglia in the subcortical white matter and internal capsule.^42^ However, the CC has been shown to be least affected by Huntington’s disease when compared with white matter that surrounds the striatum.^1^ For the purposes of this study, we chose the entire CC as the reference region, as it has (i) reasonably low Huntingtin expression (minimal direct impact of the disease), (ii) comparable TSPO expression with basal ganglia tissue (to assume similar non-specific binding) and (iii) no severe atrophy to allow meaningful analyses. In general terms, the basal ganglia and CC are anatomically and functionally distinct regions. In people with Huntington’s disease, the expression of mutant Huntingtin does not have the same impact on the various types of neurons across the brain. The assumption is that abnormal Huntingtin is a major single factor for regional neuronal damage in this disease. We are making this point to distinguish the basal ganglia from the CC regarding susceptibility to degenerative disease. The latter point refers to evidence from studies in idiopathic Parkinson’s disease.^43,44^

However, in the context of Huntington’s disease, significant atrophy in the basal ganglia^45–48^ enables the two regions of interest (caudate and putamen) susceptible to a partial volume effect. This phenomenon can introduce a bias in the activity quantification of the PET images,^49,50^ and considerably impact the validity of extracted DVRs. Here, we applied PVC to minimize the atrophy bias on our dataset. We also performed statistical testing using the uncorrected values and results remained unchanged (see Table 2). Considering the above points together, we believe that our imaging protocol delivered evaluable DVR data and the proposed analysis pipeline is reliable for this study design.

Focused on the aims of our study, it was deemed appropriate not to perform complex analyses with the laquinimod subgroups and TSPO affinity status and accept the limitation of a fixed (1-year) duration of treatment. After considering all available evidence at the set-up of the PET study, we decided to screen for TSPO affinity status and exclude low affinity binders. We acknowledge that HABs and MABs are presented together in this report and that this is a limitation of the current methodology.

In this study, Huntington’s disease patients and healthy volunteers were age matched but not for gender. Previous work in rodents have shown a greater number of microglia in females.^51,52^ This trend has been recently confirmed by ^11^C-PBR28 PET-CT data in healthy volunteers.^52^ However, with current knowledge, epidemiological and genetic sources do not highlight gender as a significant known risk factor for Huntington’s disease.^53–55^

With current evidence, the study of microglia activation in the context of manifest Huntington’s disease is a complex area for research. As neuroinflammation is a chronic process that starts in pre-manifest Huntington’s disease and develops over years, it is possible that the study of microglia activation and the effect CNS modulators (such as laquinimod) have on it require longitudinal study designs, including Huntington’s disease patients as well as pre-manifest cases.

## Conclusions

The data from the PET-CT study of the LEGATO-HD trial show that TSPO expression in the caudate and putamen does not change significantly over 1 year in patients with Huntington’s disease. In this cohort, laquinimod treatment did not improve clinical symptoms. The proposed pipeline for analysing ^11^C-PBR28 PET-CT data sets the background for future studies on neuroinflammation in Huntington’s disease.

## Supplementary Material

fcad084_Supplementary_Data

## Data Availability

The data that support the findings of this study are available from the corresponding author upon reasonable request.

## Abbreviations

CC =: corpus callosum
DD_diagn_ =: disease duration from diagnosis
DD_onset_ =: disease duration from onset
DICOM =: digital imaging and communications in medicine
DVR =: distribution volume ratio
HAB =: high affinity binder
MAB =: mixed affinity binder
MIAKAT =: molecular imaging and kinetic analysis toolbox
MPRAGE =: magnetization-prepared rapid acquisition gradient echo
PVC =: partial volume effect correction
QC =: quality control
ROI =: region of interest
SPM12 =: statistical parametric mapping, version 12
TSPO =: translocator protein
UHDRS =: unified Huntington’s disease rating scale
