# Supplementary material for: A PET-CT study on neuroinflammation in Huntington’s disease patients participating in a randomized trial with laquinimod
Source: Brain Commun. 2023 Apr 3;5(2):fcad084. doi: 10.1093/braincomms/fcad084 (PMC10069663; doi:10.1093/braincomms/fcad084)
Supplement: fcad084_Supplementary_Data [file fcad084_supplementary_data.docx]

**SUPPLEMENTARY MATERIAL**

**Supplementary Table 1. Inclusion and exclusion criteria for the LEGATO-HD PET cohort**

| **Inclusion criteria** |
| --- |
| Presence of 40-49 CAG repeats, inclusive, in the huntingtin gene based on centralized  CAG testing during screening |
| Male or female between 21-55 years of age, inclusive, with an onset of Huntington’s disease at or after 18 years of age |
| Women of child-bearing potential (women who are not post-menopausal or who have undergone surgical sterilization) must practice an acceptable method of birth control for 30 days before taking the study treatment, and 2 acceptable methods of birth control during all study duration and until 30 days after the last dose of treatment was administered. Acceptable methods of birth control in this study include: Intrauterine devices, barrier methods (condom or diaphragm with spermicide) and hormonal methods of birth control (e.g. oral contraceptive, contraceptive patch, long-acting injectable  contraceptive) |
| A sum of >5 points on the UHDRS-TMS at the screening visit |
| UHDRS-TFC ≥ 8 at the screening visit |
| Able and willing to provide written informed consent prior to any study related procedure being performed at the screening visit. Patients with a legal guardian should be consented according to local requirements. For the UK only: only patients with the capacity to give their informed consent should be enrolled |
| Willing to provide a blood sample for genomic CAG and TSPO analysis at the screening visit |
| Willing and able to take oral medication and able to comply with the study specific procedures. |
| Ambulatory, being able to travel to the study centre, and judged by the investigator as likely to be able to continue to travel for the duration of the study |
| Availability and willingness of a caregiver, informant, or family member to provide input at study visits assessing CIBIC-Plus, CDR-SB, PBA-s and HD-QoL. A caregiver is recommended to be someone who attends to the patient at least 2 to 3 times per week for at least 3 hours per occasion, and the suitability of the caregiver should be judged by the investigator |
| For patients taking allowed antidepressant medication, the dosing of medication must  have been kept constant for at least 30 days before baseline and must be kept constant  during the study |
| **Exclusion criteria** |
| Use of immunosuppressive agents, or cytotoxic agents, including cyclophosphamide and  azathioprine within 12 months prior to screening |
| Previous use of laquinimod |
| Use of moderate/strong inhibitors of CYP3A4 within 2 weeks prior to randomization |
| Use of inducers of CYP3A4 within 2 weeks prior to randomization |
| Pregnant or breastfeeding |
| Serum levels ≥3xULN of either ALT or AST at screening |
| Serum direct bilirubin which is ≥2xULN at screening |
| Creatinine clearance <60 mL/min at screening, calculated using the Cockcroft Gault  equation: (140 - age) × mass (kg) × [0.85 if female] / 72 × serum creatinine (mg/dL) × 88.4 |
| Subjects with a clinically significant or unstable medical or surgical condition that may put the patient at risk when participating in the study or may influence the results of the study or affect the patient's ability to take part in the study, as determined by medical history, physical examinations, ECG, or laboratory tests. Such conditions may include:  1. A major cardiovascular event (e.g. myocardial infarction, acute coronary syndrome, de  compensated congestive heart failure, pulmonary embolism, coronary revascularization) that  occurred during the past 6 months prior to randomization  2. Any acute pulmonary disorder  3. A CNS disorder other than HD that may jeopardize the subject's participation in the study,  including such disorders that are demonstrated on the baseline MRI (based on local read)  4. A gastrointestinal disorder that may affect the absorption of study medication  5. Renal disease  6. Cirrhotic patients with moderate or severe hepatic impairment  7. Known human immunodeficiency virus positive status. Patients will undergo an HIV test at  screening per local requirements, if applicable  8. Any malignancies, excluding basal cell carcinoma, in the 5 years prior to randomization |
| Any clinically significant, abnormal, screening laboratory result which in the opinion of the investigator, affects the patients’ suitability for the study or puts the patient at risk if he/she enters the study |
| Alcohol and/or drug abuse within the 12 months prior to screening, as defined by Diagnostic and Statistical Manual of Mental Disorders – Fourth Edition Text Revision (DSM-IV TR) criteria for substance abuse. For former alcohol and/or drug abusers, the abstinence should be confirmed by laboratory tests (drug testing and/or carbohydrate deficient transferrin (CDT) level in blood) |
| Patients with active suicidal ideation during the past month as measured by a most severe suicide ideation score of 4 (Active Suicidal Ideation with Some Intent to Act, without Specific Plan) or 5 (Active Suicidal Ideation with Specific Plan and Intent) on the baseline screening Columbia-Suicide Severity Rating Scale (C-SSRS) or subjects who answer “Yes” on any of the 5 C-SSRS Suicidal Behavior Items (actual attempt, interrupted attempt, aborted attempt, preparatory acts, or behavior) if the attempt or acts were performed within 1 year of screening, or subjects who, in the opinion of the investigator, present a serious risk of suicide |
| Patients with known intracranial neoplasms, vascular malformations, or intracranial hemorrhage |
| Known drug hypersensitivity that would preclude administration of laquinimod or placebo, such as hypersensitivity to mannitol, meglumine or sodium stearyl fumarate |
| Swallowing difficulties that would preclude administration of laquinimod or placebo capsules |
| Treatment with any investigational product within 12 weeks of screening or patients planning to participate in another clinical study assessing any investigational product during the study |
| Treatment with tetrabenazine within 30 days of the study baseline visit |
| Treatment with antipsychotic medication within 30 days of the study baseline visit |
| Women of childbearing potential. Pregnancy excluded performing pregnancy test 2days and immediately before the PET scan |
| Unsuitability for MRI (claustrophobia, metal implants) |
| Previous exposure to ionizing radiation within 12 months prior the PET scan |
| Treatment with high dose (20-30mg/daily) of benzodiazepines within 72 hours previous the PET scan |

**Supplementary Table 2. HD data: changes over time**

| Treatment | placebo (N=5) | | laquinimod (N=10) | |
| --- | --- | --- | --- | --- |
| ROIs | caudate | putamen | caudate | putamen |
| ^11^C-PBR28 *│*Δ*│*DVR | 0.34±0.45 | 0.41±0.53 | 0.16±0.09 **ns**^a^ | 0.12±0.07 **ns**^a^ |
| ^11^C-PBR28 % DVR change | 19.56±24.16 | 19.35±21.89 | 12.07±7.17 **ns**^a^ | 8.17±5.47 **ns**^a^ |

*Values are means ±1SD; Δ: delta (difference); DVR: distribution volume ratio (unitless); ns: non-significant; ^a^ comparison between baseline placebo and baseline laquinimod values*

| *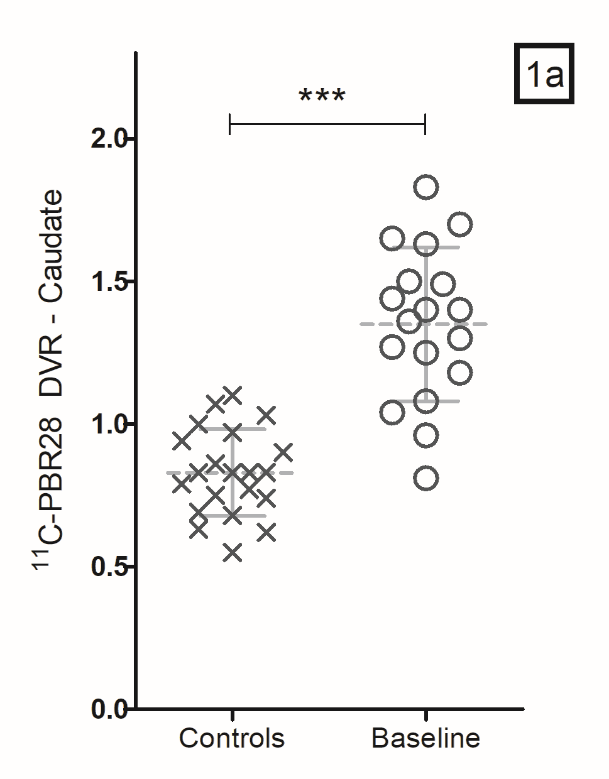* | *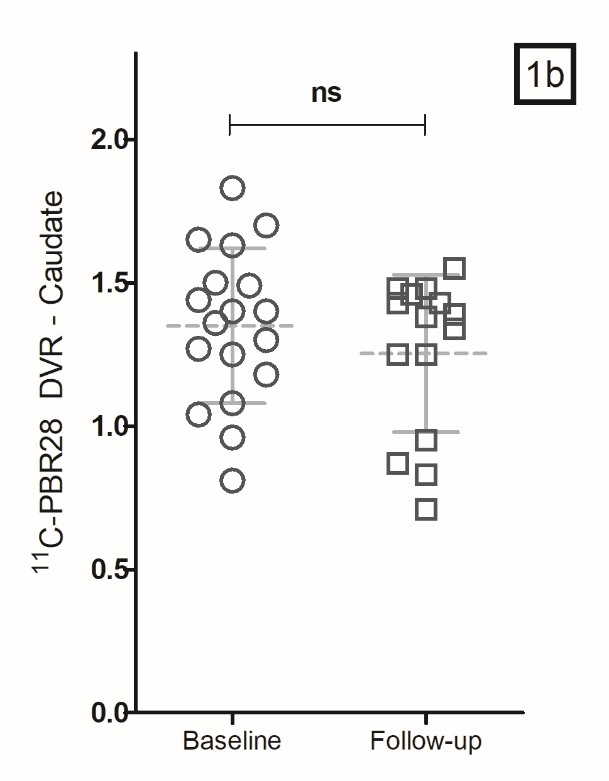* |
| --- | --- |

**Supplementary Figure 1. PET data: controls and HD patients (caudate)**

Scatter plot of 11C-PBR DVR values for caudate

**1a**. Xes: Healthy volunteers (Controls), N=21; Circles: Baseline data from HD patients, N=18, ***denotes statistical significance p<0.001 between healthy volunteers and HD patients (baseline values). Comparisons of means between controls and patients (baseline only) were performed with non-parametric Mann Whitney U tests.

**1b**. Xes: Circles: Baseline data from HD patients, N=18, Squares: Follow-up data from HD patients, N=15; ns: non-significant. Comparisons of means between baseline and follow-up DVR values (patients only) were performed with paired t-tests.

| *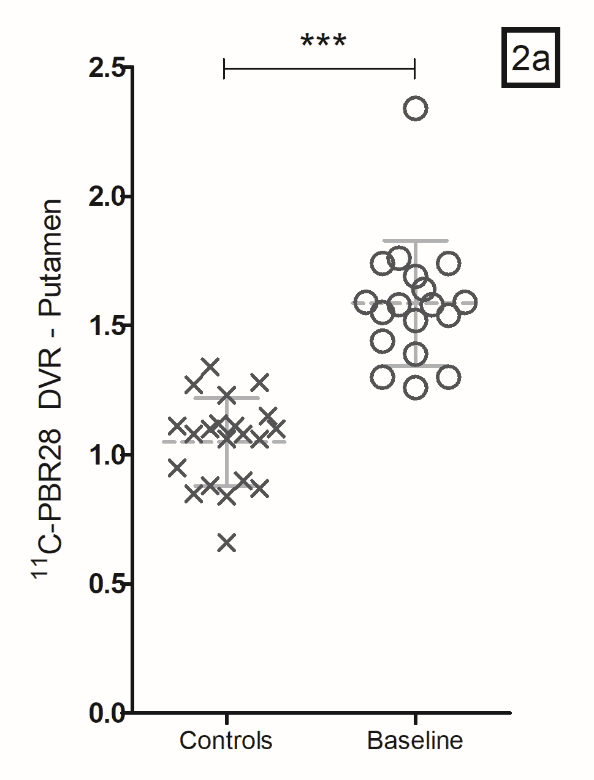* | 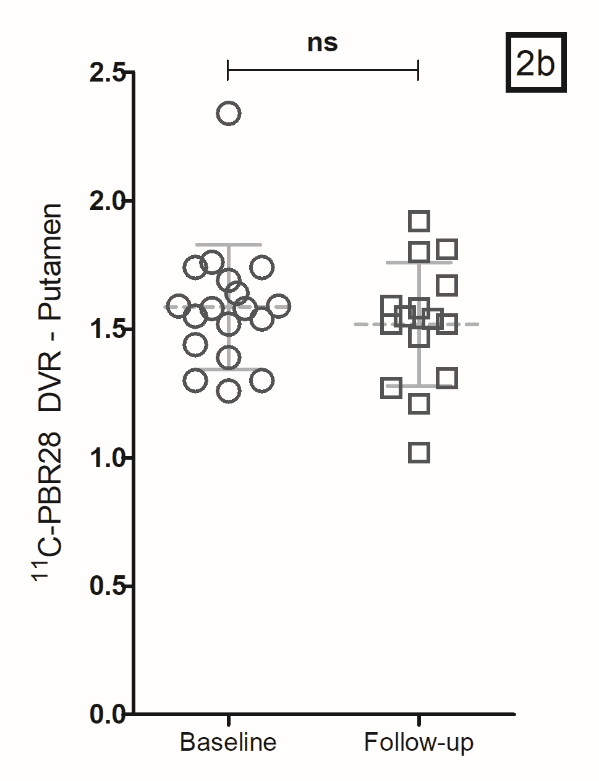 |
| --- | --- |

**Supplementary Figure 2. PET data: controls and HD patients (putamen)**

Scatter plot of ^11^C-PBR DVR values for putamen

**1a.** Xes: Healthy volunteers (Controls), N=21; Circles: Baseline data from HD patients, N=18, ***denotes statistical significance p<0.001 between healthy volunteers and HD patients (baseline values). Comparisons of means between controls and patients (baseline only) were performed with non-parametric Mann Whitney U tests.

**1b.** Xes: Circles: Baseline data from HD patients, N=18, Squares: Follow-up data from HD patients, N=15; ns: non-significant. Comparisons of means between baseline and follow-up DVR values (patients only) were performed with paired t-tests.

**
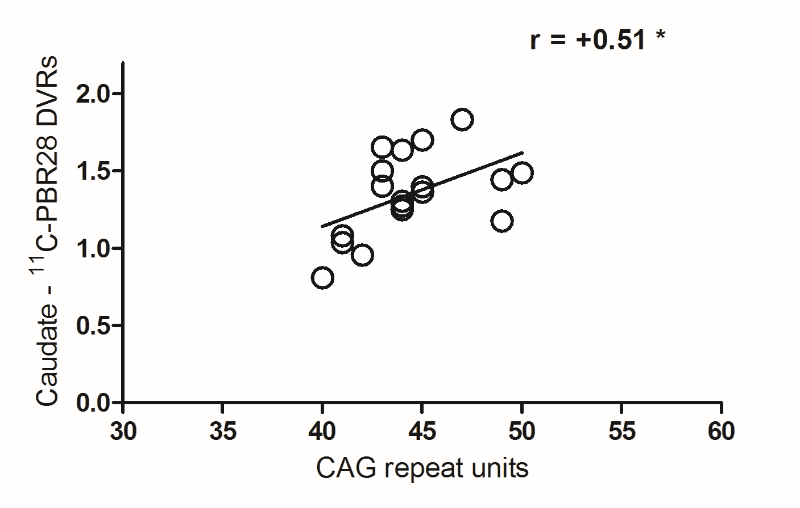
**

**Supplementary Figure 3. PET data and CAG repeats: correlation**

Spearman correlation of caudal ^11^C-PBR DVR values with CAG repeats at baseline (N=18); *denotes statistical significance p<0.05

**
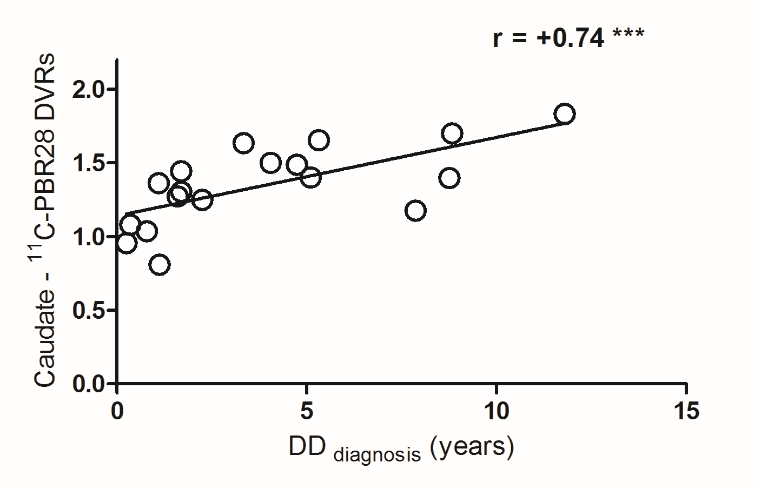
**

**Supplementary Figure 4. PET data and disease duration: correlation**

Spearman correlation of caudal ^11^C-PBR DVR values with disease duration at baseline (N=18); ***denotes statistical significance p<0.001


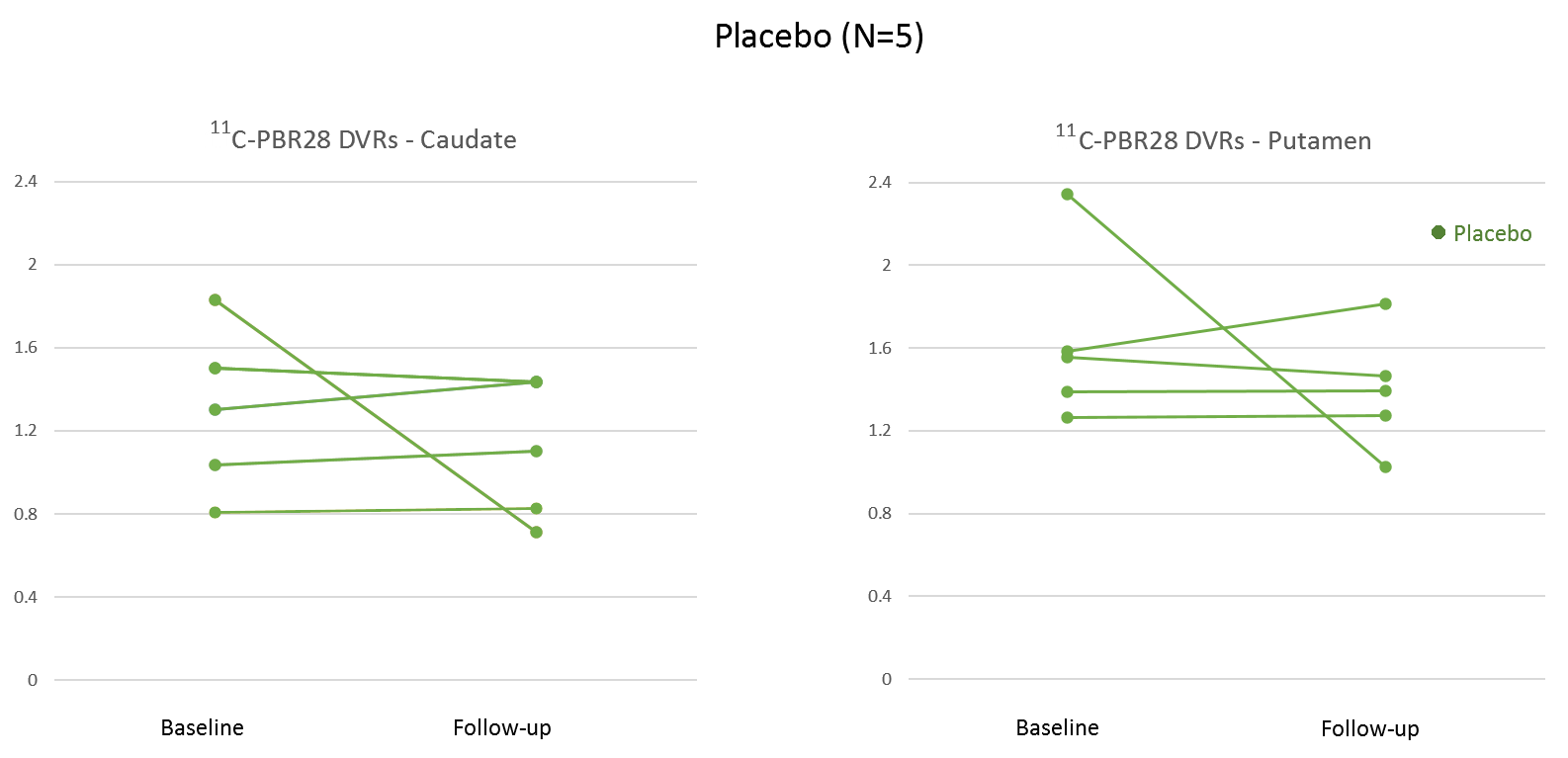


**Supplementary Figure 5. PET data: change over time (placebo)**

HD patients on Placebo (N=5). Scatter plot of ^11^C-PBR DVR values for the caudate (left) and putamen (right) connecting Baseline and Follow-up values


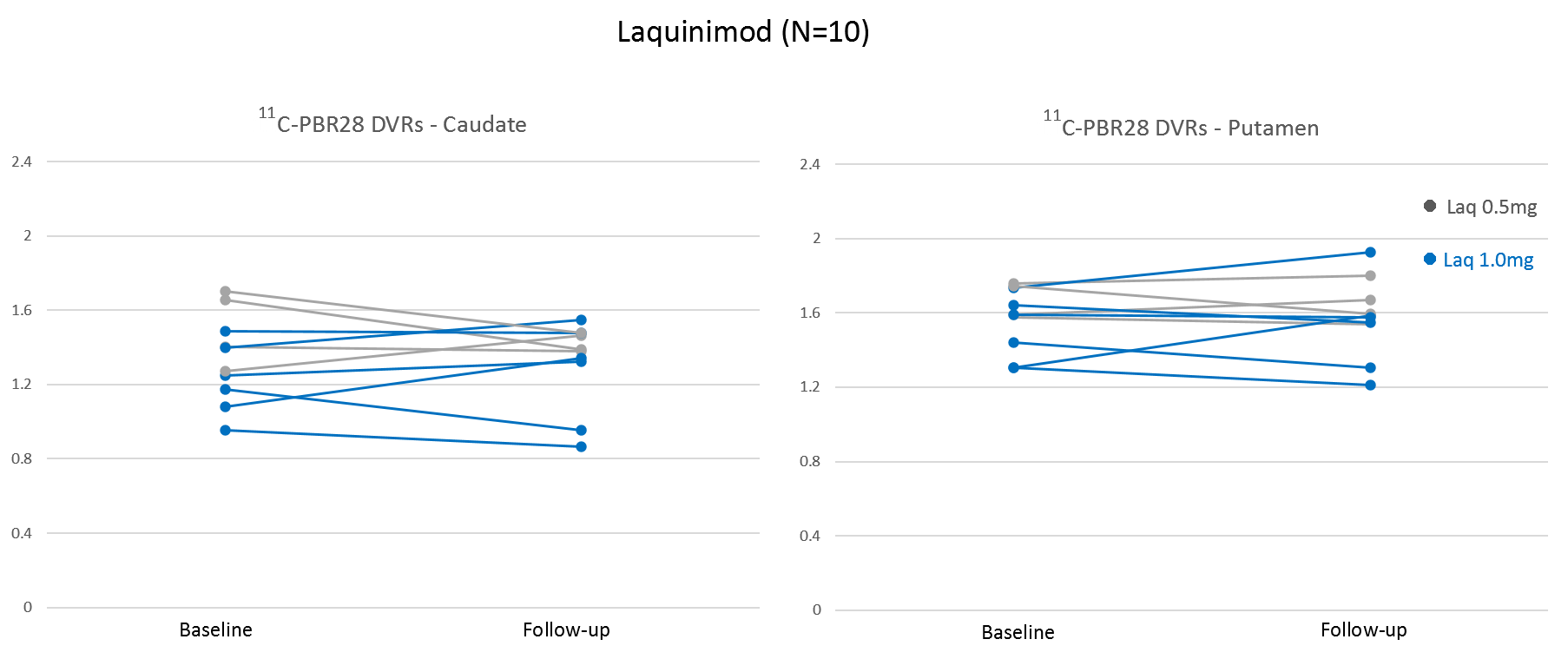


**Supplementary Figure 6. PET data : change over time (laquinimod)**

HD patients on Laquinimod (N=10). Scatter plot of ^11^C-PBR DVR values for the caudate (left) and putamen (right) connecting Baseline and Follow-up values
